# Demographic and Behavioral Correlates of Social Media Addiction and Loneliness Among Young Adults in Kyrgyzstan

**DOI:** 10.1002/brb3.71166

**Published:** 2025-12-31

**Authors:** Niyazi Ayhan, Bedir Eroğlu

**Affiliations:** ^1^ Faculty of Communication Kyrgyz‐Turkish Manas University Bishkek Kyrgyzstan; ^2^ Department of Communication Sciences Kyrgyz‐Turkish Manas University Bishkek Kyrgyzstan

**Keywords:** Kyrgyzstan, loneliness, social media addiction, social media use, young adults

## Abstract

**Introduction:**

Digital technologies now shape nearly every aspect of daily life, raising growing concern about how they influence people's psychological and social well‐being, especially among youth in developing societies. This study examines the relationship between social media addiction and perceived loneliness among young adults in Kyrgyzstan, a rapidly digitizing Central Asian country, with a particular focus on demographic differences such as age and gender.

**Methods:**

A cross‐sectional survey design was used. Data were gathered from 426 participants aged 18–26 using the Bergen Social Media Addiction Scale (BSMAS) and the UCLA Loneliness Scale. Along with correlation analysis, independent‐samples *t*‐tests, one‐way analysis of variance (ANOVA), and multiple regression analyses were conducted to examine demographic variations and relational patterns.

**Results:**

The results showed a small yet meaningful link between social media addiction and loneliness (*r* = 0.161, *p* = 0.001). Women were more likely to display higher addiction levels, whereas men reported feeling lonelier. Participants aged 24–26 exhibited the highest loneliness scores.

**Conclusion:**

These findings suggest that digital behavior and demographic factors may be important considerations in psychological screening and intervention strategies for young people.

## Graphical abstract

This study shows that young adults in Kyrgyzstan who spend more time on social media tend to feel lonelier. Even when gender and daily media use are considered, higher social media addiction is still linked to greater loneliness. The study highlights the social effects of digital behavior.

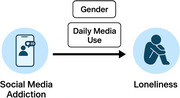

## 1 Introduction

Most people now live surrounded by digital ties that bring them closer together. For many young adults, screens have replaced much of the casual face‐to‐face conversation that once occurred. They reach out online for visibility, for reassurance, for a feeling of belonging that never seems far away. However, the same networks that link them also create distance. People may think they are close when they keep in touch online, but something quiet changes beneath the surface. The words come fast, the notifications never stop, yet the feeling often fades. Real emotion—the kind that grows when people meet—starts to thin out little by little (Turkle 2011). Some authors describe this uneasy blend of connection and distance as the “lonely crowd” of the modern world (Twenge et al. 2019). Messages appear constantly—one after another—and for a short while, they seem to bring comfort. Then, when the screen goes quiet, the quiet feels heavier than before. Messages arrive without pause, giving the impression of togetherness, yet that constant contact often leaves people more aware of their own isolation. As people return again and again to social media seeking comfort, they often find the same emptiness (Bányai et al. 2017; Elhai et al. 2021). Earlier studies link excessive social media use to loneliness, depressive symptoms, and psychological withdrawal, although cross‐sectional findings make the direction of these effects uncertain. What remains visible is a preference for brief exchanges instead of sustained human contact.

Social media addiction means that individuals uncontrollably use digital platforms. According to Kuss and Griffiths (2017) and Keles et al. (2020), this behavior usually manifests through psychological loneliness and depressive symptoms. In recent years, the link between this form of addiction and loneliness has drawn increasing interest from researchers. Some studies suggest that the relationship between social media addiction and loneliness may be both bidirectional and potentially causal. When Wang and Zeng (2024) reexamined the literature—32 studies over 20 years—they saw something familiar: Loneliness and internet addiction tend to rise together (*r* = 0.291, *p* < 0.001). Not always the same way, however. Younger people, women, and the better educated each looked different. Younger respondents differed from older ones, women from men, and education made its own mark. Even place seemed to play a part—people living in different regions did not show the same tendencies. Watching these contrasts closely can reveal which groups stand at greater risk from the digital environment. Previous studies have shown both gender‐ and age‐related differences in social media use and its addictive potential (Keles et al. 2020; Andreassen et al. 2017).

Researchers have sought to explain why social media use and loneliness tend to go hand in hand. One line of thought draws on social comparison theory (Festinger 1954; Appel et al. 2015): people look at others online and measure themselves against what they see. Most of these comparisons go upward, toward people who seem happier or more successful, which erodes self‐esteem and builds frustration. Over time, this pattern feeds loneliness, and many turn back to the same platforms to feel better. That search for comfort, however, often repeats the very feeling of isolation it tries to fix. The debate about which causes which continues, but the psychological cost is no longer in doubt.

Elhai et al. (2021) conducted a meta‐analytic review and found a similar pattern. Loneliness predicted fear of missing out (FoMO), and FoMO, in turn, was linked to problematic digital media use. In a related line of work, other researchers have noted that people who feel socially disconnected—particularly young adults—tend to use social media for distraction or to manage emotions. However, that tendency is far from universal. For many people, social media ends up being less about keeping in touch and more about getting through the day. It pulls attention away for a moment—sometimes even feels comforting—but that feeling does not last, nor does it run deep. Moreover, it leaves an uneasy but straightforward thought: maybe the digital world has not really brought us closer; maybe it has quietly changed what “being connected” means.

Demographic and personal factors may further shape this relationship. Several studies have examined the link between social media addiction and loneliness, highlighting how variables such as age, gender, and socioeconomic status can influence this association. For instance, Keles et al. (2020) reported a strong association between social media use frequency and loneliness among young adults. Examining loneliness across demographic categories helps identify vulnerable groups more effectively. Developmental and social transitions may also impact how different age or gender groups experience loneliness (Maes et al. 2019).

Although the relationship between social connectedness and loneliness has been widely studied, it remains an important topic for young adults, who undergo significant emotional and social changes. Duru (2008), for instance, found that students who felt more socially connected were less likely to feel lonely. Even though that study focused on university students, the same psychological patterns likely apply to young adults in general. These findings show that small psychological and social factors can affect how connected or how alone a person feels. Recent research has shown why loneliness and social media need closer attention, especially for adolescents and young adults. Yi et al. (2025) found that loneliness and digital disengagement reduced adolescents' participation in physical activity. Stirnberg et al. (2024) examined problematic smartphone use and noted that FoMO and depressive symptoms were strongly tied to digital routines that can both mirror and deepen social disconnection.

Boursier et al. (2020) reported that young adults who felt lonely, often along with low self‐esteem and anxiety, were more likely to use social media compulsively. However, it is still unclear whether loneliness drives this compulsive use or if excessive online activity, in turn, worsens those emotional difficulties. Nurmala et al. (2025) found that problematic social media use was associated with higher levels of mental distress among university students, especially in cross‐cultural settings such as Indonesia and Taiwan. Tung et al. (2025) reported a similar finding, noting that nomophobia mediated the link between problematic social media use and psychological distress.

In a study examining the effect of loneliness on social media addiction among university students, Savci and Aysan (2017) suggested that the findings may also be relevant for the broader young adult population. Similarly, Bekalu et al. (2019) found that the frequency of social media use is positively associated with loneliness and negatively associated with psychological well‐being. The direction of this relationship may vary depending on how and for what purposes social media is used. Balta et al. (2020) identified neuroticism and “FoMO” as potential mediators in the association between social media addiction and loneliness, although causal relationships were not confirmed.

Mitropoulou (2024) found that social media addiction was linked to loneliness. The study also showed that self‐esteem and self‐compassion played a role in this link. Earlier work suggests that this connection is still important. Wang and Zeng (2024) and Appel et al. (2015) also showed that the link can go both ways, with each side affecting the other.

Despite the growing literature, this relationship has received little attention in Central Asia. Recent research in Kyrgyzstan has examined the effects of digital transformation on children, showing new risks such as cyberbullying (Ayhan et al. 2025). Still, studies on social media addiction and loneliness among young adults in the region are limited. Central Asia, especially Kyrgyzstan, has a young population of over 30% and is rapidly digitalizing. In Kyrgyzstan, internet use reached 95% in 2024, and people aged 18–25 are the most active social media users (Datareportal 2024). Although global interest in the mental health effects of social media among young people is increasing (Boer et al. 2021), such research is still rare in this region. Earlier studies have looked at how social media motivations relate to life satisfaction among older Kyrgyz adults (Shamilova and Ayhan 2025), yet little is known about how these patterns affect younger generations. By focusing on young adults in Kyrgyzstan, this study adds a local view to the global discussion and draws attention to digital mental health issues in an underrepresented area.

The UNDP report (UNDP 2021) states that in Kazakhstan, Kyrgyzstan, Uzbekistan, Tajikistan, and Turkmenistan, young people primarily use social media passively. It also notes that restrictions on free expression, limited digital skills, and weak infrastructure are the leading causes. These factors, the report adds, may increase feelings of loneliness and digital exclusion among young people (UNDP 2021). To our knowledge, this is the first study to examine the relationship between social media addiction and loneliness in the Central Asian context. As such, scientific efforts to understand the digital experiences of youth in this region are crucial for advancing academic knowledge and informing regional policy development.

In this direction, the research aims to answer the following questions:

RQ1. Do levels of social media addiction differ according to demographic variables such as age, gender, and place of residence?
RQ2. Do levels of loneliness differ according to demographic variables such as age, gender, and place of residence?
RQ3. Is there a significant relationship between social media addiction level and the loneliness level of young adults?

## 2 Methods

This study employs a cross‐sectional and descriptive survey design to investigate the relationship between social media addiction and loneliness among young adults. Descriptive research seeks to identify and explain existing phenomena without manipulating variables, allowing researchers to observe what is naturally present (Creswell 2014).

### 2.1 Participants

This study uses a convenience sampling method to examine the relationship between social media addiction and loneliness. Convenience sampling is an effective technique that increases generalizability and minimizes selection bias in research. The sampling process was carried out in public areas such as libraries, youth centers, and parks in the city of Bishkek between December 11, 2024 and January 20, 2025. Participants were approached at various times of day to ensure diversity in the sample, and those who met the inclusion criteria and voluntarily agreed to participate were included in the study. This method ensures that the sample accurately represents the universe by giving each individual an equal chance of being selected and strengthening the findings' generalizability (Berndt 2020).

The study targeted young adults aged 18–26. This age group is the age group that uses social media most intensively and is an ideal sample group to examine the relationship between loneliness and social media addiction. Individuals in this age range are at the peak of social media use, and loneliness can be felt more intensely during this period (Primack et al. 2017).

### 2.2 Measurement

The study used quantitative data collection methods, and the following instruments were employed to measure the relevant constructs: (1) a demographic information form, (2) the Bergen Social Media Addiction Scale (BSMAS), and (3) the UCLA Loneliness Scale (ULS).

### 2.3 Bergen Social Media Addiction Scale

BSMAS developed by Andreassen et al. (2012) was used to measure the participants' social media addiction levels. The scale includes six items to measure the frequency and duration of social media use and the effects of social media use on an individual's social, academic, and personal life. Participants respond to each item on a 5‐point Likert scale (1 = never, 5 = always). In the original study of the scale, the internal consistency coefficient (Cronbach's alpha) was reported as *α* = 0.89.

The BSMAS was translated into Kyrgyz using a forward–backward translation method. First, two bilingual experts translated the original English version into Kyrgyz. Then, an independent translator who was blind to the original version translated the Kyrgyz version back into English. The two versions were compared to ensure semantic equivalence. A pilot study with 30 participants was conducted to test clarity and cultural appropriateness. Necessary adjustments were made on the basis of the feedback before the main data collection. The internal consistency of the Kyrgyz version was acceptable (*α* = 0.76), indicating good reliability.

### 2.4 UCLA Loneliness Scale

To measure loneliness levels, the ULS, developed by Russell (1996) and one of the most widely used tools in the loneliness literature, was applied. The scale contains 20 items and was developed to measure individuals' feelings of loneliness, levels of social isolation, and quality of interpersonal interaction. The original form has a 4‐point Likert‐type structure (1 = never, 4 = very often). However, in this study, the scale was adapted to a 5‐point Likert‐type (1 = never, 5 = always) to enhance consistency across all instruments used in the analysis. When applied carefully, this kind of modification can improve distributional balance and analytical consistency without affecting the instrument's psychometric quality (Sullivan and Artino 2013).

The scale was translated into Kyrgyz using a forward–backward translation method involving two independent bilingual experts. First, the scale was translated from English to Kyrgyz by a native‐speaking translator with a background in psychology. Then, a second bilingual expert, blinded to the original version, back‐translated the Kyrgyz version into English. Discrepancies were reviewed and resolved through agreement. A small pilot study with 30 participants was conducted to test clarity, cultural relevance, and item understanding. Minor wording changes were made in response to the feedback. The final Kyrgyz version showed high internal consistency (*α* = 0.91), confirming its linguistic and conceptual validity.

### 2.5 Data Analysis Process

Previous studies in the field have shown that a sample size of approximately 400 is sufficient to detect small to medium effect sizes in correlational and regression analyses with adequate statistical power (Cohen 1992; Field 2013). Therefore, the sample size of this study (*N* = 426) was considered adequate to support the planned statistical analyses.

The data collected within the scope of the research were analyzed using SPSS (Statistical Package for the Social Sciences) 23.0 software. First, descriptive statistics (frequency, percentage, mean, standard deviation) were used to describe the demographic characteristics of the participants. In line with the study's primary purpose, Pearson correlation analysis was applied to examine the relationship between social media addiction and loneliness levels. This analysis allows us to determine the strength and direction of the linear relationship between two continuous variables. In addition, a *t*‐test for independent samples was used to evaluate the effect of dichotomous variables such as gender on the level of social media addiction. One‐way analysis of variance (ANOVA) was applied to examine the differences between three or more groups (e.g., different age groups) within the sample. Before conducting ANOVA tests, the assumption of homogeneity of variances was checked using Levene's test. For group comparisons, effect sizes were also calculated using Cohen's *d* to assess the practical significance of statistically significant mean differences.

The results confirmed that this assumption was met, supporting the validity of the comparisons across demographic subgroups. The internal consistency of the scales used was evaluated, and Cronbach's alpha coefficients showed that both scales had a high level of reliability. In addition, the sample size (*N* = 426) was sufficient to support the validity of the statistical analyses.

### 2.6 Demographic Characteristics of Study Participants

Demographic characteristics of 426 individuals who participated in the study are presented in Table 1. According to the age distribution, 35.9% (*n* = 153) were between the ages of 18 and 20, 31.2% (*n* = 133) between the ages of 21 and 23 and 32.9% (*n* = 140) between the ages of 24 and 26. In terms of the type of residence, 46.7% (*n* = 199) of the participants lived in the village for a long time, 16.7% (*n* = 71) in the district and 36.6% (*n* = 156) in the city. According to their economic status, 4.7% (*n* = 20) of the participants had low income, 89.4% (*n* = 381) had medium income, and 5.9% (*n* = 25) had high income. When the duration of daily social media use was evaluated, 4.0% (*n* = 17) of the participants stated that they used social media for 0–1 h, 20.0% (*n* = 85) for 1–3 h, 48.6% (*n* = 207) for 3–5 h, and 27.5% (*n* = 117) for more than 5 h. According to the results of self‐assessment of social media addiction, 18.8% (*n* = 80) of the participants stated that they were addicted, 63.1% (*n* = 269) were partially addicted, and 18.1% (*n* = 77) were not addicted.

**TABLE 1:** Demographic characteristics of the participants (*N* = 426).

Variable	Category	*n*	%
Gender	Female	228	53.5
	Male	198	46.5
Age group	18–20	153	35.9
	21–23	133	31.2
	24–26	140	32.9
Place lived longest	Village	199	46.7
	District	71	16.7
	City	156	36.6
Perceived economic status	Low	20	4.7
	Middle	381	89.4
	High	25	5.9
Daily social media use	0–1 h	17	4.0
	1–3 h	85	20.0
	3–5 h	207	48.6
	More than 5 h	117	27.5
Self‐perceived social media addiction	Yes	80	18.8
	Partially	269	63.1
	No	77	18.1

## 3 Findings

In this section, the findings related to social media addiction and loneliness levels, which are the main variables of the study, are presented. First, both variables' descriptive statistics (mean, standard deviation, minimum, and maximum values) are reported. Then, whether social media addiction and loneliness levels show a significant difference according to demographic variables such as gender, age group, place of residence, and economic status was analyzed by independent samples *t*‐test and one‐way ANOVA. Finally, the relationship between social media addiction and loneliness was evaluated by Pearson correlation analysis.

### 3.1 Findings on the Level of Social Media Addiction

This section examines whether social media addiction levels show significant differences according to demographic variables. For this purpose, independent samples *t*‐test, and one‐way ANOVA were applied. As a result of the independent samples *t*‐test conducted according to the gender variable, a significant difference was observed in social media addiction scores, *t*(424) = 2.19, *p* = 0.029, Cohen's *d* = 0.213. The mean score of female participants (*M* = 2.96, SD = 0.77) was higher than that of male participants (*M* = 2.80, SD = 0.74). This difference has a small effect size. In the ANOVA analysis conducted according to age groups, no significant difference was found between the groups, *F*(2, 423) = 2.614, *p* = 0.074. The highest mean was observed in the 18–20 age group (*M* = 2.98), but the difference was not statistically significant. Therefore, no post‐hoc analysis was performed. No significant difference was found in the study conducted according to the places where the participants lived for a long time, *F*(2, 423) = 1.082, *p* = 0.340. The social media addiction scores of the village, district, and city groups were at similar levels. No significant difference was found between the groups in the ANOVA conducted according to the perceived economic status, *F*(2, 423) = 1.362, *p* = 0.257. Although the mean of the low‐income group was high (*M* = 3.28), this difference was not statistically significant. No significant difference was observed between the social media addiction scores of the participants according to their daily social media usage time, *F*(3, 421) = 0.654, *p* = 0.581. Although the 0–1 h usage time group had the highest mean, the difference did not reach statistical significance. Finally, no significant difference was found between the social media addiction scores of the participants according to their perception of themselves as social media addicts, *F*(2, 423) = 0.827, *p* = 0.438. When these findings are evaluated in general, it is seen that there is a significant difference in social media addiction levels only according to the gender variable. No significant differences were observed regarding other demographic variables (Table 2).

**TABLE 2:** Independent variable *t*‐test and analysis of variance (ANOVA) analysis results of social media addiction on demographic characteristics (*N* = 426).

Independent samples *t*‐test								
**Variable**	Group	*N*	Mean	Std. deviation	Std. error	*t*	Sig. (2‐tailed)	Cohen's *d*
Gender	Female	228	2.9605	0.771	0.051	2.192	0.029	0.213
	Male	198	2.7997	0.736	0.052			
ANOVA								
**Variable**	Group	*N*	Mean	Std. deviation	*df*	*F*	Sig.	
Age group	18–20	153	2.979	0.769	2 423	2.614	0.074	
	21–23	133	2.774	0.757				
	24–26	140	2.889	0.740				
Place lived longest	Village	199	2.877	0.790	2 423	1.082	0.340	
	District	71	2.784	0.837				
	City	156	2.942	0.675				
Perceived economic status	Low	9	3.277	0.978	2 423	1.362	0.257	
	Middle	392	2.872	0.755				
	High	25	2.953	0.716				
Daily social media use	0–1 h	17	2.980	0.696	3 421	0.654	0.581	
	1–3 h	85	2.892	0.816				
	3–5 h	206	2.919	0.717				
	More than 5 h	117	2.806	0.80049				
Self‐perceived social media addiction	Yes	80	2.787	0.77721	2 423	0.827	0.438	
	Partially	269	2.907	0.74427				
	No	77	2.913	0.79224				

### 3.2 Findings Regarding Loneliness Levels

This section analyzes whether loneliness levels show significant differences according to demographic variables. Independent sample *t*‐test and one‐way ANOVA were applied in the analyses.

As a result of the independent sample *t*‐test conducted according to the gender variable, a statistically significant difference was found in the levels of loneliness, *t*(424) = −5.52, *p* < 0.001, Cohen's *d* = −0.536. The average loneliness score of male participants (*M* = 2.80, SD = 0.45) was significantly higher than that of female participants (*M* = 2.55, SD = 0.50). This difference has a medium effect size. According to the ANOVA analysis conducted on age groups, a significant difference was found between the groups, *F*(2, 423) = 3.70, *p* = 0.025. The lowest level of loneliness was observed in the 18–20 age group (*M* = 2.59), and the highest level was observed in the 24–26 age group (*M* = 2.75).

After the significant ANOVA result, the Tukey HSD post hoc test revealed that loneliness levels differed statistically significantly among all age groups (*p* < 0.001). These findings indicate that the feeling of loneliness may increase with age. In the analysis conducted according to the places where the participants lived for a long time, no significant difference was found between loneliness levels, *F* (2, 423) = 0.955, *p* = 0.386. Loneliness scores were similar among the village, district, and city groups. In the ANOVA conducted according to the perceived economic status, no significant difference was observed between the groups, *F*(2, 423) = 0.791, *p* = 0.454. Although the average loneliness level of the low‐income group was high, the difference was not statistically significant. No significant difference was found between loneliness scores for daily social media usage time, *F*(3, 421) = 0.805, *p* = 0.492. Finally, no significant difference was found between loneliness scores according to self‐perception of social media addiction, *F*(2, 423) = 0.329, *p* = 0.720 (Table 3).

**TABLE 3:** Analysis of *t*‐test and analysis of variance (ANOVA) results for loneliness by demographic characteristics (*N* = 426).

Independent samples *t*‐test								
**Variable**	Group	*N*	Mean	Std. deviation	Std. error	*t*	Sig. (2‐tailed)	Cohen's *d*
Gender	Female	228	2.546	0.502	0.033	−5.515	0.001	−0.536
	Male	198	2.804	0.454	0.032			
ANOVA								
**Variable**	Group	*N*	Mean	Std. Deviation	*df*	*F*	Sig.	
Age group	18–20	153	2.588	0.531	2 423	3.704	0.025	
	21–23	133	2.672	0.488				
	24–26	140	2.745	0.454				
Place lived longest	Village	199	2.682	0.471	2 423	0.955	0.386	
	District	71	2.592	0.455				
	City	156	2.679	0.544				
Perceived economic status	Low	9	2.872	0.555	2 423	0.791	0.454	
	Middle	392	2.661	0.489				
	High	25	2.670	0.583				
Daily social media use	0–1 h	17	2.800	0.456	3 421	0.805	0.492	
	1–3 h	85	2.638	0.510				
	3–5 h	206	2.647	0.474				
	More than 5 h	117	2.703	0.531				
Self‐perceived social media addiction	Yes	80	2.706	0.512	2 423	0.329	0.720	
	Partially	269	2.654	0.487				
	No	77	2.666	0.517				

### 3.3 The Relationship Between Social Media Addiction and Loneliness

In this section, Pearson correlation analysis was performed to examine the relationship between social media addiction and loneliness. The analysis results showed a positive, weak, and significant relationship between the two variables (*r* (426) = 0.161, *p* = 0.001).

This finding shows that as social media addiction increases, loneliness also tends to increase. However, the low correlation coefficient reveals that the strength of this relationship is limited. Although the two variables increase together, the level of the relationship remains weak (Table 4).

**TABLE 4:** Pearson correlation between social media addiction and loneliness (*N* = 426).

Variables	1	2
**1. Social media addiction**	1	0.161**
**2. Loneliness**	0.161**	1

**Correlation is significant at the 0.01 level (2‐tailed).

The results of the multiple regression analysis indicated that the overall model was statistically significant, *F*(6, 418) = 8.675, *p* < 0.001, explaining approximately 11.1% of the variance in loneliness levels (Adjusted *R* ^2^ = 0.098). Among the predictors, social media addiction (*β* = 0.196, *p* < 0.001) and gender (*β* = 0.266, *p* < 0.001) were significant positive predictors of loneliness. In contrast, age, place of residence, perceived economic status, and daily social media use were not statistically significant predictors (*p* > 0.05). These findings suggest that higher levels of social media addiction and being male are associated with increased loneliness among young adults in this sample (Table 5).

**TABLE 5:** Multiple linear regression analysis predicting loneliness (dependent variable: loneliness (UCLA Loneliness Scale [ULS] total score)).

Predictor variable	*B*	SE	*β*	*t*	*p*
(Constant)	1.781	0.236	—	7.560	<0.001
**Social media addiction**	0.128	0.030	0.196	4.205	<0.001
**Gender**	0.264	0.049	0.266	5.430	<0.001
**Age**	0.042	0.029	0.070	1.434	0.152
**Place of residence**	0.002	0.026	0.003	0.072	0.942
**Perceived economic status**	−0.037	0.082	−0.021	−0.452	0.652
**Daily social media use**	0.040	0.029	0.064	1.365	0.173

## 4 Discussion

In this study, female participants reported higher levels of social media addiction than male participants. This suggests that gender may influence how people use social media. Some studies have found that women are generally more active in creating and sharing content on social or relational topics, which could partly explain their higher use (Al‐Menayes 2015). Other research indicates that women often use social media for emotional support and social connection, which may contribute to addictive behaviors (Andreassen et al. 2017). Recent studies support this view. For instance, Blackwell et al. (2017) found that women showed stronger motives for relationship maintenance and emotional release in their social media use, and that these motives were linked to addictive patterns. Likewise, Kuss and Griffiths (2017) reported that social media addiction is more common among women, possibly due to social comparison, need for approval, and desire for connection. However, not all findings agree. In some cultural contexts, men tend to display riskier online behaviors, which may also increase addiction risk (Hou et al. 2017). Therefore, gender differences should be interpreted in light of cultural and motivational factors.

The study found that male participants reported higher levels of loneliness than female participants (*p* < 0.001, *d* = −0.536). This finding is consistent with earlier research showing that men may be more prone to social isolation. For example, Maes et al. (2019) observed that men are generally less likely than women to seek social support, which may increase their sense of loneliness.

In addition, apparent differences in loneliness levels emerged across age groups, with loneliness rising steadily with age. The highest loneliness scores were seen among participants aged 24–26, which may reflect reduced social support, growing personal responsibilities, and identity changes that occur toward the end of young adulthood. Hawkley and Cacioppo (2010) stated that loneliness increases with age, beginning in young adulthood, due to psychosocial factors. These results are in line with Erikson's theory of psychosocial development. The theory says that people aged 18–40 are in the “intimacy versus isolation” stage, and not forming close relationships often leads to loneliness (Kemph 1969). High levels of loneliness among people aged 24–26 may reflect weaker emotional ties or greater inner pressure at this stage of life. Other demographic factors, such as residence, perceived economic status, and daily time spent on social media, did not significantly affect loneliness levels, suggesting that psychosocial rather than contextual factors are the main drivers of loneliness.

According to the study's findings, there is a positive but weak yet statistically significant relationship between social media addiction and loneliness (*r* = 0.161, *p* = 0.001). This result is consistent with previous studies showing that excessive social media use can increase loneliness (Bányai et al. 2017; Savci and Aysan 2017). In particular, social media addiction may lead individuals to withdraw from face‐to‐face interactions and engage in social comparison, contributing to feelings of inadequacy.

Andreassen et al. (2012) revealed that social media addiction can cause deterioration in individuals' social relationships and an increase in feelings of loneliness. These results show that the relationships individuals establish through social media do not replace fundamental social interactions and cannot fill emotional gaps. Social Displacement Theory can also explain this relationship. According to this theory, as individuals spend more time online rather than in offline social interactions, their social ties weaken and their loneliness increases (Kraut et al. 1998).

Multiple regression analysis was used to test whether social media addiction predicts loneliness after controlling for demographic variables, including age, gender, place of residence, perceived economic status, and daily social media use. The overall model was statistically significant (*F*(6, 418) = 8.67, *p* < 0.001) and explained about 9.8% of the variance in loneliness (adjusted *R* ^2^ = 0.098). Among all variables, social media addiction (*β* = 0.196, *p* < 0.001) and gender (*β* = 0.266, *p* < 0.001) were significant predictors. Higher social media addiction and being male were both linked to greater loneliness. Age, perceived economic status, daily social media use, and place of residence did not show significant effects.

These findings are consistent with earlier research showing that social media addiction can be an independent psychosocial risk factor for loneliness, regardless of demographic background (Kırcaburun and Griffiths 2018; Turel and Serenko 2012). The results also align with the Compensatory Internet Use Theory, which argues that individuals may turn to problematic online behaviors—such as excessive social media use—to compensate for unmet offline emotional or social needs (Kardefelt‐Winther 2014). However, this coping strategy may ironically worsen feelings of disconnection rather than alleviate them.

Similar findings were reported in previous research. Kırcaburun and Griffiths (2018) found a significant relationship between social media addiction and loneliness, showing that loneliness may work both as a cause and as a result of social media use. Turel and Serenko (2012) likewise noted that as social media addiction increases, individuals tend to experience greater social isolation, which in turn may contribute to heightened loneliness.

Although the association between social media addiction and loneliness has been studied in various cultural settings, Kyrgyzstan offers a distinct context due to its transitional societal structure. Factors such as collectivist family values, limited mental health infrastructure, and passive patterns of social media use—shaped by concerns around political expression—may influence how young people experience loneliness. Additionally, digital inequality and rapid urbanization may intensify disconnection in a society still balancing traditional community ties with digital modernity. Therefore, these cultural and structural factors may shape both the prevalence and expression of social media‐related loneliness in ways that differ from more frequently studied Western contexts.

In conclusion, although social media addiction appears to be a significant predictor of loneliness, its effect is modest. It should be examined within more comprehensive models that reflect the multifaceted nature of loneliness.

## 5 Results

The findings indicate a statistically significant but modest association between social media addiction and loneliness among young adults in Kyrgyzstan. Correlation analysis revealed a weak yet significant positive relationship (*r* = 0.161, *p* = 0.001). However, the explained variance was low (*R* ^2^ = 0.026), suggesting that other factors contribute to loneliness beyond digital behavior (see Table 2 for detailed statistics).

Demographic differences also emerged. Female participants reported higher levels of social media addiction, whereas male participants experienced greater loneliness. In addition, participants aged 24–26 showed the highest loneliness scores (see Tables 3 and 4 for detailed comparisons).

Finally, multiple regression analysis confirmed that social media addiction significantly predicted loneliness even after controlling for demographic variables (*F*(6, 418) = 8.67, *p* < 0.001; adjusted *R* ^2^ = 0.098). Higher levels of social media addiction and being male were both associated with greater loneliness. In contrast, age, residence, perceived economic status, and daily social media use were not significant predictors (see Table 5).

## Author Contributions

**Niyazi Ayhan**: conceptualization. **Niyazi Ayhan, Bedir Eroğlu**: methodology. **Niyazi Ayhan**: formal analysis. **Niyazi Ayhan, Bedir Eroğlu**: investigation. **Bedir Eroğlu**: resources. **Bedir Eroğlu**: data curation. **Niyazi Ayhan**: writing – original draft. **Niyazi Ayhan, Bedir Eroğlu**: writing – review and editing. **Niyazi Ayhan**: visualization. **Niyazi Ayhan**: supervision. **Niyazi Ayhan**: project administration.

## Funding

The authors have nothing to report.

## Data Availability

The data that support the findings of this study were collected through a structured questionnaire and analyzed using SPSS. These data are available from the corresponding author, Dr. Niyazi Ayhan, upon reasonable request. Data sharing is possible provided that participant confidentiality and ethical standards are upheld.
